# Deep Endometriosis of the Terminal Ileum Presenting With Cyclic Recurrent Small Bowel Obstruction

**DOI:** 10.7759/cureus.45636

**Published:** 2023-09-20

**Authors:** Kristal Ha, Devon Marks, Robert D Bennett, Emad Mikhail

**Affiliations:** 1 Obstetrics and Gynecology, University of South Florida Morsani College of Medicine, Tampa, USA; 2 Surgery/Colon and Rectal Surgery, University of South Florida Morsani College of Medicine, Tampa, USA

**Keywords:** intestinal endometriosis, endometriosis diagnosis, endometriosis surgery, endometriosis, bowel endometriosis, deep infiltrating endometriosis (die), ileal endometriosis, laparoscopic surgery for endometriosis, endometriosis and chronic pelvic pain, endometriosis excision

## Abstract

Here, we discuss a case of a 42-year-old premenopausal female who presented with chronic pelvic pain and recurrent small bowel obstruction during menstruation. The patient reported a nine-year history of pelvic pain and a four-year history of episodic small bowel obstruction requiring multiple prior inpatient admissions. During these admissions, the obstruction was managed conservatively with bowel rest and nasogastric tube placement; however, symptoms would recur with subsequent menstrual cycles. Computed tomography showed diffusely dilated loops of small bowel with a transition point in the central anterior pelvis, and magnetic resonance enterography revealed a mass-like area involving small bowel loops in the mid pelvis. The patient underwent laparoscopic surgical intervention including bowel resection with re-anastomosis, hysterectomy, bilateral salpingectomy, and left oophorectomy. Intraoperative findings included severe distention of the proximal bowel with a discrete deep endometriosis lesion of the terminal ileum which was confirmed on final pathologic examination. This case emphasizes the importance of considering endometriosis as the etiology of recurrent catamenial small bowel obstruction, particularly in premenopausal women.

## Introduction

Endometriosis is a complex disease defined by the presence of endometrial-like tissue outside of the uterus. It is a chronic inflammatory condition associated with pelvic pain and infertility most commonly in reproductive-age women [1]. Three phenotypes of endometriosis have been described: peritoneal endometriosis, ovarian endometriosis, and deep endometriosis (DE). DE nodules extend more than 5 mm beneath the peritoneum and have been described in pelvic and extra-pelvic locations [2]. DE of the bowel has been estimated to affect 3.8-37% of patients with endometriosis, with the most frequent sites of bowel involvement being the sigmoid colon, rectum, ileum, appendix, and cecum [3]. DE of the small intestine has been estimated to represent less than 5% of DE lesions [2]. Studies have found that less than half of patients with endometriosis of the small bowel have a preceding diagnosis of pelvic endometriosis [4]. Patients typically report colicky abdominal pain which prompts workup for gastrointestinal processes and results in a significant delay between symptom onset and definitive diagnosis [5]. Diagnosis is often delayed due to variable presentation, symptom overlap with other more common conditions, and the need for surgery for definitive diagnosis [1].

## Case presentation

**Figure 1:**
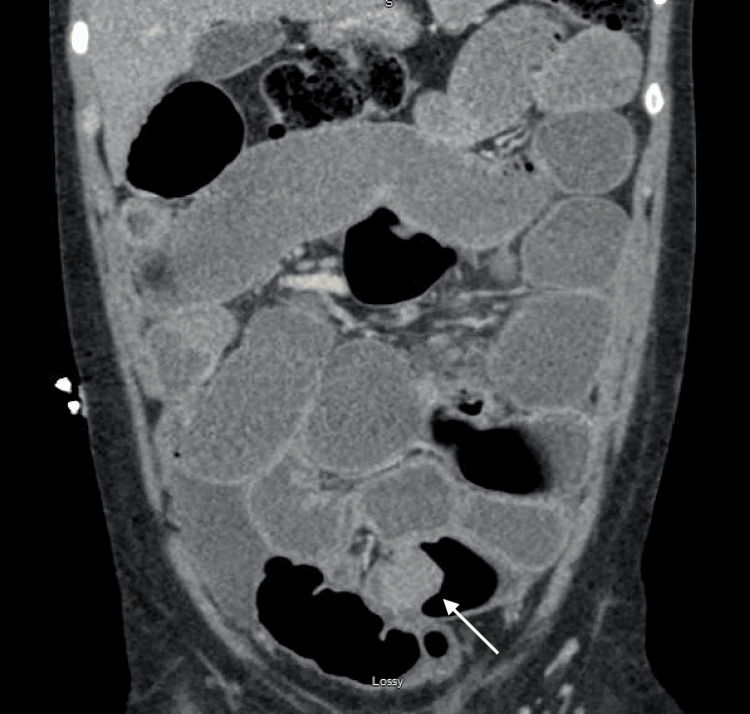
CT scan demonstrating diffusely dilated small bowel loops proximal to a transition point with associated enhancing nodularity (arrow) in the central anterior pelvis.

**Figure 2:**
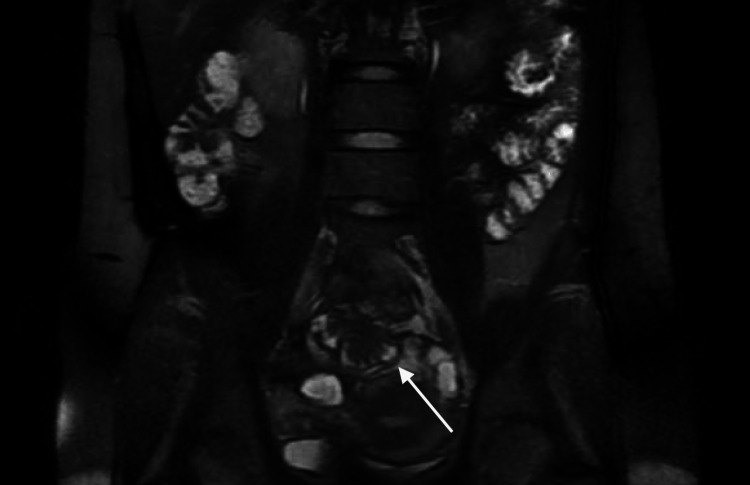
MR enterography with coronal T2-weighted image demonstrating infiltrating mass-like tissue (arrow) along a loop of distal small bowel with associated luminal narrowing.

**Figure 3:**
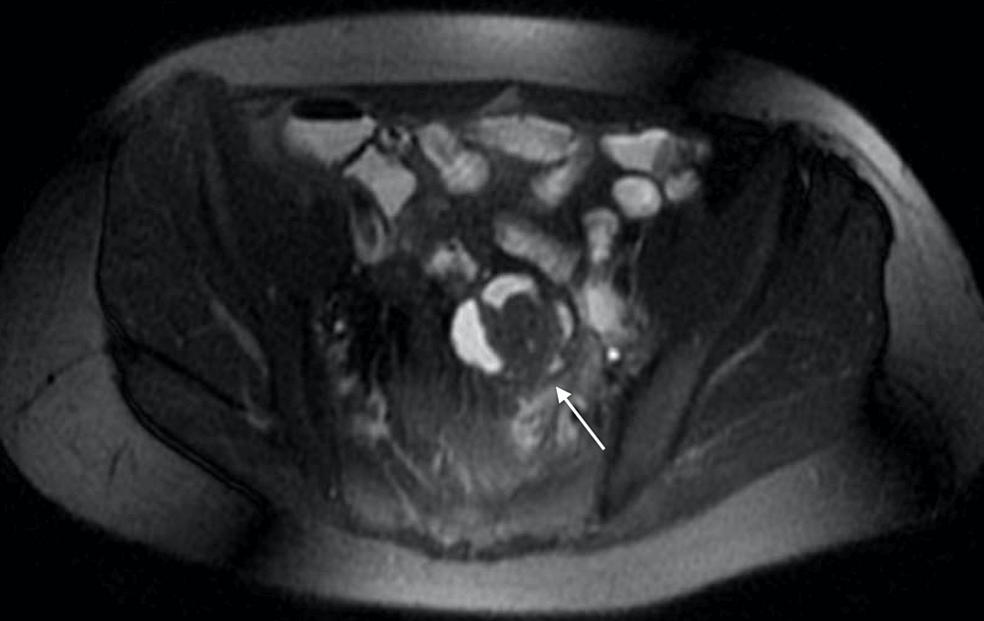
MR enterography with the axial T2-weighted image demonstrating infiltrating mass-like tissue (arrow).

**Figure 4:**
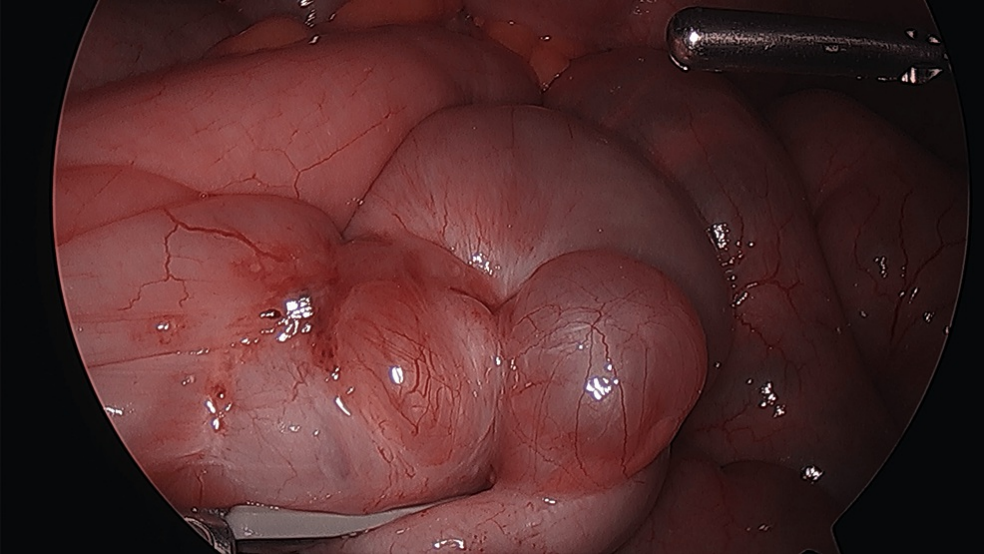
Laparoscopic image of the discrete deep endometriosis lesion of the terminal ileum.

**Figure 5:**
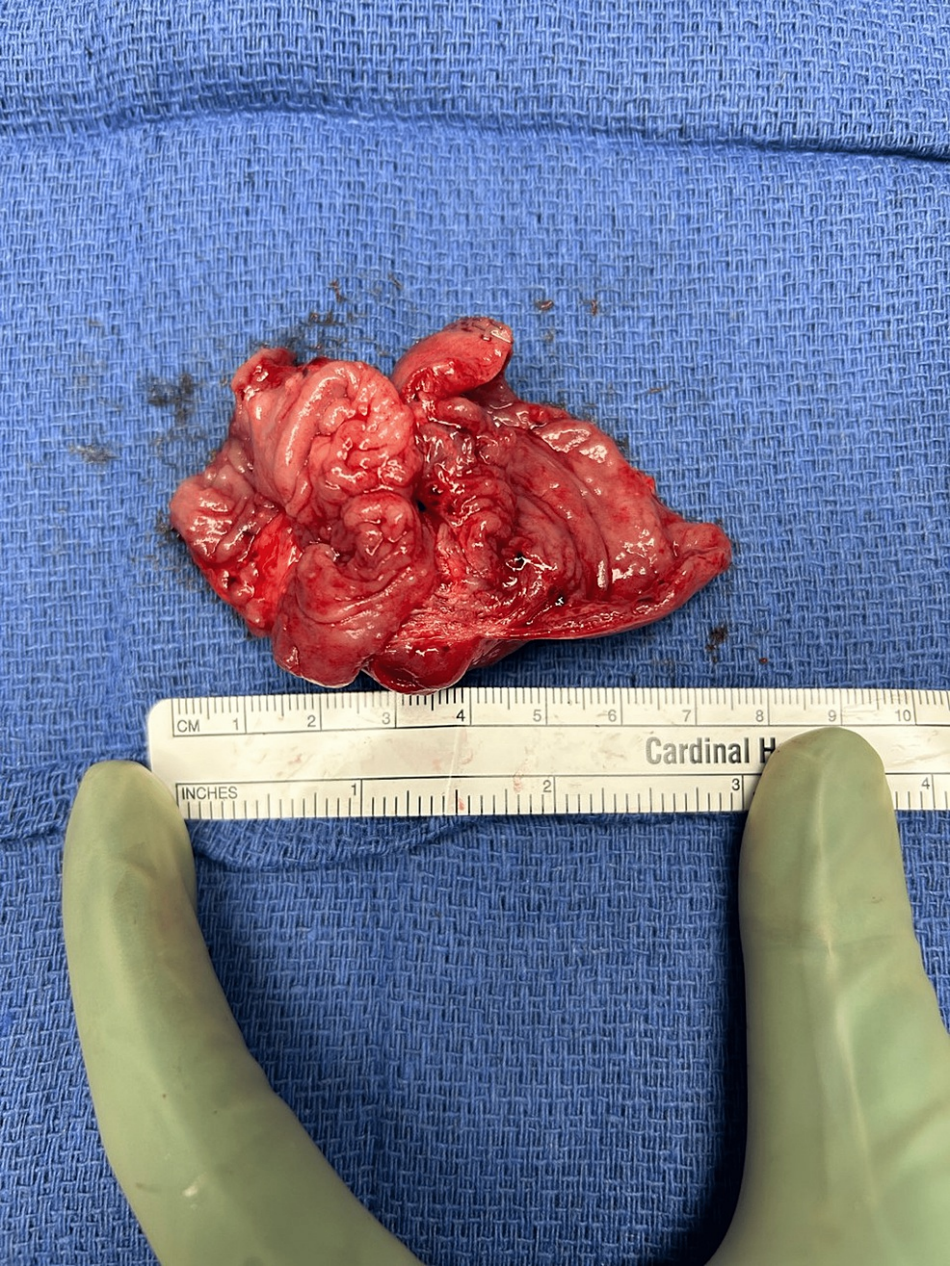
Resected portion of small bowel with the associated isolated deep endometriosis lesion.

A 42-year-old G0P0 female with a known history of endometriosis presented for evaluation of chronic pelvic pain in the setting of recurrent small bowel obstruction. The patient reported a nine-year history of pelvic pain for which she previously underwent laparoscopy at an outside facility, the documentation of which was not available at the time of consultation, and was reportedly diagnosed with endometriosis. She subsequently attempted medical management with multiple hormonal therapies including combined oral contraceptives, gonadotropin-releasing hormone (GnRH) analogues, and progestin-only pills; however, she was unable to achieve symptom relief. At the time of presentation, the patient had not been on hormonal therapy for two years. She had also developed recurrent small bowel obstruction requiring multiple inpatient admissions during the preceding four years. During these admissions, conservative management with bowel decompression with nasogastric tube and bowel rest with NPO status resulted in relief of obstruction, but these episodes were recurrent with her subsequent menstrual cycle. Computed tomography (CT) scan showed diffusely dilated loops of small bowel with a transition point in the central anterior pelvis (Figure 1). Prior magnetic resonance (MR) enterography of the pelvis revealed a T2 hypointense mass-like area involving small bowel loops in the mid pelvis spanning 3.1 cm (Figures 2, 3). The patient underwent combined laparoscopic surgical intervention with gynecology and colorectal surgery, at which time intraoperative findings included severe distention of the proximal bowel with a discrete deep endometriosis lesion of the terminal ileum (Figures 4, 5). The remainder of her small bowel was systematically evaluated both visually and manually and no other lesions were identified throughout the intestine. Laparoscopic small bowel resection with primary anastomosis was performed in addition to hysterectomy, bilateral salpingectomy, left oophorectomy, and excision of endometriosis. Final pathology revealed endometriosis of the terminal ileum involving the muscularis propria, endometriosis of bladder peritoneum, right periureteric endometriosis, focal involvement of endometriosis of bilateral fallopian tubes, adenomyosis, and left endometrioma. The patient’s postoperative course was uncomplicated, and she was deemed stable for discharge home on postoperative day 9.

## Discussion

The diagnosis of endometriosis is complicated by variations in patient presentation and poor correlation between phenotype, location of lesions, and symptoms. Additionally, response to treatment is highly variable and appears to differ based on the endometriosis subtype. While the recommended management of peritoneal endometriosis is less clear, surgical excision is generally the treatment of choice for endometriomas and DE [1]. Surgery for DE of the bowel usually consists of bowel resection and anastomosis, full-thickness disc excision, and/or shaving of superficial lesions. Meta-analysis of 49 studies examining surgical treatment of bowel endometriosis estimated an overall endometriosis recurrence rate of about 10%, with a recurrence rate of only 5.8% in those who underwent bowel resection with anastomosis [2]. The most common locations of bowel DE are the rectum, rectosigmoid junction, and sigmoid colon. Given that small bowel endometriosis makes up less than 5% of DE, it is an extremely rare cause of small bowel obstruction [2]. Our case therefore represents a rare and unique presentation of DE and highlights the importance of considering endometriosis in the differential diagnosis of recurrent cyclic small bowel obstruction in reproductive-age females. Treatment of bowel DE presents a unique challenge as it requires gynecologic expertise in endometriosis as well as anatomical and surgical expertise in bowel surgery. This interplay between specialties typically requires a multidisciplinary approach which may contribute to delay in diagnosis and treatment. Currently, there are no studies specifically examining the recurrence of small bowel endometriosis; therefore, further data specific to this rare presentation would assist in patient counseling and surgical planning.

## Conclusions

DE of the small bowel is a rare phenomenon that requires a high level of clinical suspicion. Clinicians should consider bowel DE as a possible etiology of small bowel obstruction in premenopausal patients, particularly in the setting of chronic pelvic pain, dysmenorrhea, or known endometriosis. Additional workups may include transvaginal or transrectal ultrasound as well as magnetic resonance imaging and/or enterography. Increased clinical awareness and imaging may decrease delays in diagnosis and surgical management thereby reducing patient morbidity and improving quality of life.
